# A Snapshot of Peer Relationships in Children and Youth: Pre- Versus During COVID-19

**DOI:** 10.3390/ijerph21121552

**Published:** 2024-11-25

**Authors:** Jordyn Manis, Shannon L. Stewart

**Affiliations:** Faculty of Education, University of Western Ontario, 1137 Western Rd, London, ON N6G 1G7, Canada; sstewa24@uwo.ca

**Keywords:** COVID-19, peer conflict, social relationships, children and youth, interRAI ChYMH

## Abstract

Strong peer relationships are an essential component of a healthy, happy, and long lifetime. Given that there is little understanding of the impact of COVID-19 on peer relationships, this study explored the effects of the COVID waves on peer relationships for clinically referred children and youth while controlling for age, sex, and income. 11,281 children and youth between the ages of 4 and 18 years, who were receiving services from mental health agencies across Ontario from January 2018–March 2022, were assessed using the interRAI ChYMH. Chi-square tests of independence and logistic regression analysis was performed. Overall, as expected, there were significantly fewer peer relationship difficulties during COVID-19 compared to the pre-pandemic period. Despite the general decline of peer issues, individuals between the ages of 8–18, particularly males, were more likely to experience peer relationship difficulties compared to those 4–7 years old. Additionally, children from the lowest income households experienced greater peer relationship difficulties during COVID-19 when compared to those from the highest income households. The findings from this study demonstrate the nuanced changes in social behaviours due to the ongoing pandemic for children and youth and highlight those youth who are most in need of social and behavioural interventions.

## 1. Introduction

Supportive social relationships with peers are an essential component of a positive quality of life [1]. For children and youth in particular, good quality peer relationships have important effects on adjustment, development, and psychological well-being [1,2]. The extant literature has shown that healthy peer relationships promote resilience by encouraging a sense of affiliation and offering ways of coping, particularly for those who are socio-economically vulnerable [1]. Friendship support is not only an immediate predictor of resilient psychosocial functioning, but when cultivated during adolescence, its benefits carry into adulthood [3]. Taken together, the research reveals that children who are accepted by their peer group and who have strong friendships tend to fare better overall [4].

### 1.1. The Importance of Peer Relationships

For preschoolers, peer relationships are characterized by reciprocity, positivity and make-believe play, high levels of social interaction, and appropriate conflict resolution [5]. Additionally, both an understanding of emotion and individual temperament are associated with socially competent peer play [6]. Children who display positive play behaviours are more widely accepted by their peers and tend to have more reciprocal friendships [7]. Conversely, those without friends remain at a considerable developmental disadvantage [5,8]. Even at a young age, developmental status affects the extent to which children are socially accepted within the preschool or play-group context [9]. This is because children with developmental challenges often experience significant difficulties with aspects of peer-related social competence [5,10]. Considering that these individuals are more likely to have poorer status among their peers, it is important to investigate peer-related differences for this high-risk group across different age ranges.

Establishing stable relationships with peers remains important through middle childhood as feeling connected to others greatly contributes to children’s overall well-being [11]. Specifically, friendships at this age are essential for providing comfort and camaraderie, building social skills, and protecting against victimization [12].

Peer relationships are of particular importance during adolescence as it is during this period that youth tend to rely more on their friends for support [1,13,14,15,16]. At this stage, youth are largely concerned with status and popularity, and consistently seek peer acceptance [17]. As a result, peer relationships have a greater influence on many areas of functioning, including school engagement, academic performance, and disruptive behaviours [18]. Research has shown that close peer relationships in adolescence is associated with positive health outcomes, including decreased depression, anxiety, and suicidal ideation [15,19]. Further, having a single, supportive close friend has been linked with fostering resilience processes in vulnerable adolescents, as this relationship allows individuals to find meaning and strength amidst considerable adversity [1]. However, other researchers have demonstrated that particular friendships can have a negative impact on adjustment [20]. Specifically, peers with certain characteristics, such as being anti-social or depressed, can influence others in detrimental ways [2]. Thus, only healthy, high-quality friendships are beneficial for the psychological adjustment of youth.

### 1.2. Peer Relationships in COVID-19

The COVID-19 pandemic has created extraordinary challenges for children and youth with regard to their peer relationships. During the initial lockdown period, parents were primarily concerned with the lack of opportunities for social interaction [21]. A study conducted by AlHarbi and colleagues [22] found that, according to their parents, children between the ages of one and eighteen had fewer friends during the pandemic and lockdown periods compared to pre-pandemic rates. Further, research suggests that many experienced conflicts with friends as a result of differing opinions regarding adhering to the COVID-19 restrictions and methods of coping [23]. However, to date, there is a lack of sufficient evidence regarding the impact of COVID-19 on social behaviours in children and youth.

For preschoolers, kindergarten is the primary space for social interaction. However, during lockdown, their social relations were primarily limited to close family members [24]. Considering that routines are essential for the overall functioning of children at this age, their mental health and well-being may have been compromised by the sudden impact of isolation and loss of contact with peers [25]. However, preliminary evidence surrounding the effects of COVID-19 on preschoolers’ social development is mixed. For instance, while some researchers have found a decline in young children’s social skills after the onset of the pandemic (e.g., [26]), Quinones and Adams [27] observed that two preschoolers were able to maintain a friendship via digital technologies and the virtual world.

In a study conducted by Caduff [28], the majority of middle school students reported experiencing negative changes in their peer interactions and friendships both during and after temporary school closures, including significant changes in the nature of their relationships, notable differences in peer interactions, and enduring changes in social-emotional temperament resulting from their peer interactions. In the same study, only a small group of female students identified positive changes in their peer interactions during the pandemic. Several researchers have found that, throughout COVID-19, children sought ways of connecting with their friends, but these methods were less effective compared to typical in-person interactions [29,30,31]. However, some researchers have found that higher technology use for friendship maintenance purposes buffered against feelings of self-isolation and diminished the impact of COVID-19 on friendship quality [32]. Carter and colleagues [30] also found that the children who struggled to maintain friendships amid the tight restrictions generally experienced deep feelings of separation, loss, and anger. Further, the higher the child’s pre-pandemic peer social skills, the less likely they were to act out, exhibit fearfulness, or demonstrate anxious withdrawal behaviours [33]. Interestingly, bullying among peers appeared to decline during the lockdown period when schooling was remote across all forms of bullying [34], with the exception of cyberbullying [35]. Specifically, elementary school students were at greater risk of experiencing cyberbullying and victimization during the pandemic [36], particularly in Asian countries and Australia [37]. Additional research points to a decline in cyberbullying in Western countries [37]. Further evidence is needed to confirm whether these trends remained stable once children were able to return to school.

During the COVID-19 pandemic, many youth encountered an increase in peer and conduct problems [38]. In fact, not being able to spend time with friends was one of the primary difficulties for the majority of youth [39,40]. Similarly to younger children, the majority attempted to maintain regular online contact with friends; while some youth found it did not have the same benefits as in-person interactions and reported it was challenging and lonely [40,41], others reported having stable peer relationships during the lockdown period, which protected against poorer psychological outcomes [42,43]. A study conducted by Houghton and colleagues [44] found that having stronger pre-COVID friend support predicted greater levels of positive mental health during the pandemic. Further, internalizing problems prior to the pandemic were predictive of greater friend support during COVID-19 [45]. Unsurprisingly, greater social support was also associated with decreased feelings of loneliness and COVID-related distress [46]. Conversely, both traditional bullying victimization and perpetration increased during the pandemic, specifically for males and lower secondary school students [47]. However, for some youth, periods of school closures acted as a protective factor for peer difficulties, where students felt safer staying home [48]. Bullying rates dropped significantly during the period of remote learning in 2020 (e.g., [35,49,50]), offering students who typically experience peer difficulties and victimization a stretch of relief [51]. This is partially due to the 6-foot distancing measures required as well as lockdown restrictions. In another study, measures of perceived stress declined during lockdown periods for youth who were bullied [52]. Evidently, the experience of youth during COVID-19 varied greatly [53,54]; research examining the specific experience of various population sub-groups is crucial in order to gain a complete understanding of the nuanced outcomes of the pandemic.

### 1.3. Peer Relationships and Sex

Sex is a powerful component in shaping and nurturing the behaviours of children and youth, particularly those that transpire within the peer context [4]. From a young age, children tend to form relationships with same-sex peers [55], and there are notable differences in terms of the content of play [4]. Boys tend to excel in managing friendships in a broader social context, responding to peer transgressions, and creating fun and excitement amongst one another [56,57]. Conversely, for girls, friendships are a strong source of emotional and motivational support, particularly during difficult times [1,57]. Furthermore, while girls tend to seek out dyadic friendships, feel more empathy for others and maintain connection-oriented goals within the peer context, boys tend to hold more self-beneficial goals, such as solidifying their own dominant position within the group [58]. Research also suggests that adolescent girls are more attached to their peers than males [59], and they perceive a stronger correlation between popularity and social competence [60].

Overall, limited research exists on the topic of sex-related differences in peer relationships for children and youth during COVID-19. However, one study found that boys appeared to experience higher rates of peer victimization, but lower levels of mental health difficulties when compared to girls [61]. Findings from another study revealed that girls were more heavily impacted by the limited interactions with peers compared to boys [62]. 

### 1.4. Peer Relationships in Lower-Income Populations

Children in marginalized sub-populations may be especially challenged when it comes to interpersonal relationships due to their experiences of trauma, involvement with public systems of care, and mental health difficulties in the home [63]. In particular, children and youth from low-income households often endure a variety of barriers to creating meaningful connections [64]. They often enter school with fewer social skills and rely on their peers to develop communication, conflict resolution, stress management, coping, and problem-solving abilities [64]. However, research has shown that children and youth from disadvantaged families experience higher rates of victimization and rejection [65,66,67].

Not surprisingly, marginalized youth have been disproportionately affected by the COVID-19 pandemic due to pre-existing inequalities and thus require special attention [68]. Specifically, youth from lower socioeconomic backgrounds experienced less access to peer support than their higher-income peers [69,70]. A study conducted by Stevens and colleagues [71] also found a small but significant increase in peer relationship problems for disadvantaged youth during the pandemic compared to the pre-pandemic period. Interestingly, younger people and those with higher subjective socioeconomic status reported more negative impacts on their friendships, particularly feeling lonelier and more isolated, compared to older counterparts [72]. 

### 1.5. Current Study

In these unprecedented times, there is a growing need to understand the lingering effects of the COVID-19 pandemic on children and youth. This study examined peer relationships among clinically referred children and youth across three age groups as well as between the different waves of COVID-19. Whether certain subgroups of children were disproportionately affected was also examined (e.g., sex, income). To date, this is the first study to investigate the impact of the pandemic on children’s peer relations across the different waves, following the initial onset of the pandemic in March 2020, in Canada.

Based on the extant literature, it is hypothesized that (1) there will be a relationship between the waves of COVID-19 and peer relationship difficulties. Specifically, there will be a decline in peer relationship difficulties following the onset of COVID-19. Secondly, (2) age will be related to peer relationship difficulties such that those in middle childhood and adolescence will experience more peer problems than younger children. Thirdly, (3) given that males tend to struggle more overtly with relationship difficulties, sex will be related to peer relationship difficulties, with males experiencing greater social challenges than females. Finally, it is expected that (4) income will be related to peer relationship difficulties, with children and youth from the lowest income experiencing the greatest social difficulties, both pre- and during COVID-19.

## 2. Materials and Methods

### 2.1. Participants

The sample included 11,281 clinically referred children and youth between the ages of 4 and 18 years, who were receiving services from mental health agencies across Ontario in the years leading up to the pandemic or during the pandemic until March 2022. Participants were divided into three different age categories, in accordance with previous studies [73,74,75], to identify potential age-related differences across the sample. The age categories were 4 to 7 years old, 8 to 11 years old, and 12 to 18 years old. Furthermore, sex categories of male and female were established. Data from individuals who identified in the other category was excluded from analysis (*n* = 71). All participants were assessed using the *interRAI Child and Youth Mental Health Assessment* as part of their standard care to evaluate areas of functioning, needs, risks, and resilience [76].

### 2.2. Measures

#### 2.2.1. interRAI ChYMH

The interRAI ChYMH is a comprehensive, standardized, psychometrically sound assessment instrument intended for clinical use across various mental health service sectors [76]. It is a needs-based assessment tool that is composed of over 400 clinician-rated items, divided into 22 subsections, and addresses a variety of domains related to child and youth mental health (e.g., academic, social, family relations), thus providing a thorough, all-inclusive summary of needs. The interRAI ChYMH is embedded with unique scales and algorithms intended to provide further information on symptom intensity and promote evidence-based clinical decision making in specific areas of functioning. Several studies have demonstrated rigorous reliability and validity across these scales and algorithms, particularly in relation to child and youth populations (Cronbach’s alpha greater than 0.70 for all scales, except the Sleep Scale which is 0.66) [77,78,79,80,81,82,83,84,85].

#### 2.2.2. Peer Relationships Index

The peer relationships index, embedded within the interRAI ChYMH, measures the level of conflict within an individual’s peer group and is used to assess friendship formation in the current study. The index consists of five items reflective of friendship dynamics, including social inclusion by peers (this item is inversely scored), conflict with or repeated criticism of close friends, friends are persistently hostile or critical of child/youth, pervasive conflict with peers (excluding close friends), and peer group included individuals with persistent antisocial behaviour. All items are coded with scores of either 0 or 1, with zero indicating no issue and one indicating the child has experienced the behaviour in question within the last three days. Total scores on the peer relationships index range from 0 to 5, where higher scores indicate greater risk of conflict with peers. In this study, the peer relationships index was dichotomized to assign strong peer relationships for total scores of 0–1 and difficulties with peer relationships for total scores of 2 and above. This was carried out based on a specific cut point to aid in variables to simplify the results.

### 2.3. Procedures

Data for this study were collected through semi-structured interviews guided by the ChYMH, as well as through additional information obtained from multiple sources, including, but not limited to, the child themselves, the parent and/or legal guardian, and other mental health professionals. Further details on the child were collected through direct observation by the scoring clinician, as well as through a review of available clinical documents, to construct a comprehensive viewpoint of the child’s strengths and areas of need. All agencies obtained consent from the families and the child/youth as part of standard of practice. In line with the licencing of the use of interRAI instruments, de-identified, electronic data were shared with interRAI, subject to existing laws on confidentiality and data use. Data from Canadian-based agencies were entered into a deidentified web-based software system for secure storage on the interRAI Canada server for research and quality assurance. TSPS 2 guidelines for secondary data analyses were met through this process and the University of Western’s Research Ethics Board provided approval for the analysis of the data utilized in this research study.

Data used in this study were drawn from assessments completed two years prior to the pandemic (January 2018–February 2020) and during the pandemic (March 2020–March 2022). The data were further divided into six time periods, corresponding to a pre-pandemic period of 26 months, and the first five waves of the COVID-19 pandemic (see Ontario Agency for Health Protection and Promotion, 2023). Waves indicate distinct intervals where provincial cases increased and declined. The wave boundaries, also consistent with other studies (e.g., [86]), were as follows: Wave 1 ranged from the beginning of March 2020, when the pandemic was declared, until the end of August 2020, Wave 2 was defined as September 2020 until February 2021, Wave 3 as March 2021 until July 2021, Wave 4 as August 2021 until December 2021, and Wave 5 as December 2021 until March 2022. Participants assessed using the ChYMH during these timeframes were included in this study.

Median Household Income was also calculated for each participant and used as a determinant of socioeconomic status [87]. Three-digit postal codes were matched to 2016 Census data from Statistics Canada [88] to obtain each participant’s median household income after tax. For this study, the household income variable was divided into four categories in line with previous research [86]. The categories were <$57,367, $57,367 to $70,334, $70,335 to $84,750, and >$84,750.

### 2.4. Data Analytic Approach

Demographic data were collected through preliminary analyses. Pearson’s chi-square tests of independence were used to investigate peer relationship difficulties across the five waves of COVID-19, to identify differences between age groups and sex, and to determine whether lower income populations were disproportionately struggling with peer relationships. Finally, to gain a clearer picture of what occurred during the various waves of COVID-19, hierarchical logistic regression analysis was performed. Factors identified as significant from the analyses above, including waves, age, sex, and household income, were used as independent variables, while peer relationships acted as the dependent variable. Results were interpreted with a significance value of *p* ≤ 0.001. SAS 9.4 was used for all analyses. The assumptions for all tests were followed to control for threats to statistical conclusions.

## 3. Results

### 3.1. Preliminary Analyses

Chi-square tests were used to determine the rates of various characteristics related to the sample (i.e., sex, age, average household income) across the pre-pandemic period and each of the pandemic waves. Please see Table 1 for demographic characteristics.

### 3.2. Bivariate Analyses

Several chi-square tests of independence were conducted to identify potential differences in peer relationships pre- and during COVID-19 with respect to different variables. Firstly, chi-square tests of independence were conducted to investigate the various patterns of peer relationship difficulties throughout the different waves of COVID-19. The findings revealed that, compared to the pre-pandemic period, children and youth were significantly less likely to experience peer relationship difficulties during COVID-19 (χ^2^(5) = 98.933, *p* < 0.001) with small effects (Cramer’s *V* = 0.094). When compared to the pre-pandemic period, children and youth were more likely to experience fewer peer difficulties in Wave 2 (χ^2^(1) = 25.368, *p* < 0.001; Cramer’s *V* = −0.060), Wave 3 (χ^2^(1) = 47.921, *p* < 0.001; Cramer’s *V* = −0.084), Wave 4 (χ^2^(1) = 30.590, *p* < 0.001; Cramer’s *V* = −0.067), and Wave 5 (χ^2^(1) = 30.442, *p* < 0.001; Cramer’s *V* = −0.069). No significant difference was found between the pre-pandemic period and Wave 1. According to Hypothesis 1, it was expected that peer relationship difficulties would decline following the onset of COVID-19. Hypothesis 1 was partially supported. Specifically, findings revealed that while peer relationship difficulties began to decline in Wave 2, there was no significant difference between the pre-pandemic period and Wave 1.

Next, a chi-square test of independence was conducted to compare the status of peer relationships across three age groups both pre- and during COVID-19. The age groups were: 4–7 years old, 8–11 years old, and 12–18 years old. While there was no significant difference in terms of peer relationships across the age groups pre-COVID (see Table 2), the findings revealed that during COVID, children in middle childhood (ages 8–11) and adolescence (ages 12–18) were significantly more likely to have poorer peer relationships than younger children (χ^2^(2) = 18.442, *p* < 0.001) with small effects (Cramer’s *V* = 0.057). As seen in Table 3, 8.5% of children between the ages of 4 and 7 experienced peer difficulties during COVID-19, whereas 14.2% of children between the ages of 8 and 11 and 14.4% of adolescents between the ages of 12 and 18 experienced peer difficulties. These findings support Hypothesis 2, which stated that older children and youth would experience more peer problems than younger children.

Chi-square tests of independence were also conducted to identify potential differences between males and females with respect to their peer relationship difficulties. Findings revealed that males (χ^2^(1) = 38.138, *p* < 0.001) with small effects (Cramer’s *V* = −0.058) were significantly more likely to have peer relationship difficulties (19%) than females (14.7%). Hence, Hypothesis 3, which suggested that males would have more peer problems than females, was supported.

Finally, chi-square tests of independence were utilized to determine whether those in lower-income households were disproportionately struggling in terms of their peer relationships. Findings revealed that children and youth in the 1st income quartile (χ^2^(1) = 24.973, *p* < 0.001) with small effects (Cramer’s *V* = −0.066) were significantly more likely to have peer relationship difficulties (19%) than children and youth in the 4th income quartile (14.3%). Similarly, children and youth in the 2nd income quartile (χ^2^(1) = 20.434, *p* < 0.001) with small effects (Cramer’s *V* = −0.063) were significantly more likely to have peer relationship difficulties (18.8%) than children and youth in the 4th income quartile. These findings support Hypothesis 4, which stated that children and youth from the lowest-income households would experience the greatest social challenges, both pre- and during COVID-19.

### 3.3. Multivariate Analysis

Hierarchical multivariate logistic regression analysis was used to predict peer relationship difficulties using age, sex, household income, and waves as predictors. The youngest age, the male sex, the first income quartile, and the pre-pandemic period was used as the reference group in the analysis. Assumptions were tested and all were met. The first model included waves as the sole predictor, with peer relationship difficulties as the dependent variable. The second model also included age as a predictor variable, with peer relationship difficulties remaining as the dependent variable. Sex was added as a predictor variable in model three, and model four included household income as a final predictor. See Table 4 for model fit information. Notably, although the model improved at every step, the final model only accounts for roughly less than 2% of the dependent variable, suggesting the need for other predictors in future research.

Results indicated that the first model was significant (χ^2^ = 101.900, *df* = 5, *p* < 0.001). Contrary to what was hypothesized, children and youth experiencing peer relationship difficulties did not differ when comparing the pre-pandemic period versus during the first wave of COVID. However, as expected, children and youth had lower odds of experiencing peer relationship difficulties during Wave 2 of the pandemic compared to the pre-pandemic period. Also as expected, children and youth had lower odds of experiencing peer relationship difficulties during the other waves compared to the pre-pandemic period. Model two, which included age, was also significant (χ^2^ = 127.560, *df* = 7, *p* < 0.001). Controlling for waves, compared to children aged 4–7 years, 8–11-year-old children had higher odds of experiencing peer relationship difficulties. Similarly, compared to 4–7-year-olds, youth aged 12–18 years had higher odds of experiencing peer difficulties. The third model, which included sex, was significant (χ^2^ = 160.222, *df* = 8, *p* < 0.001). As predicted, controlling for age and waves, females had lower odds than males of experiencing peer relationship difficulties. The final model was also significant, suggesting that the four predictors, when taken together, reliably differentiate between those who experienced peer relationship difficulties and those who did not (χ^2^ = 190.496, *df* = 11, *p* < 0.001). As expected, controlling for age, sex, and waves, children and youth in the 4th quartile had lower odds of experiencing peer relationship difficulties compared to those in the lowest quartile. However, children and youth in the 2nd income quartile and 3rd income quartile did not differ from those in the 1st income quartile. Overall, this model accurately predicts 59.8% of youth who experienced peer relationship difficulties. Therefore, there is a need to examine other independent variables that may predict peer relationship difficulties in the future. See Table 5 for more information.

## 4. Discussion

The influence of the COVID-19 pandemic on children and youth has been robust and widespread [89]. While lack of social interaction during the initial waves of COVID-19 was identified as a potential area of need [21], little research has been conducted to explore the complexities of the social impacts resulting from this period of isolation. This study aimed to address the gap in the literature by conducting an in-depth examination of peer relationships pre- versus during COVID-19, identifying unique patterns across different age groups, time periods, and population subgroups. Overall, as predicted, there were significantly fewer peer relationship difficulties during COVID-19, compared to the pre-pandemic period, for clinically referred children and youth.

### 4.1. Peer Relationship Difficulties Across COVID-19 Waves

The results yielded an interesting pattern of social behaviours over the course of the pandemic. Specifically, while 20% of children and youth experienced peer relationship difficulties prior to COVID-19, 17% experienced difficulties with peers during Wave 1, 14.2% during Wave 2, 11.6% during Wave 3, 13.4% during Wave 4, and 11.7% during Wave 5. It is possible that environmental factors such as social distancing restrictions lifting and periods of school closures may explain fluctuations over time. For instance, the majority of schools in Ontario, and across Canada, were able to safely reopen in September of 2020 (Wave 2) and again in September of 2021 (Wave 4). Consequently, according to the findings from this study, rates of friendship difficulties rose slightly during those periods of in-person schooling; peer relationship difficulties were at their lowest during periods of mandated school closures.

Surprisingly, there were no significant differences in peer relationship difficulties before the onset of the pandemic and in Wave 1. It is possible that many children and youth, even as young as seven, quickly adapted and began to interact virtually with others [27,90], providing more online opportunities for peer engagement and conflict. Secondly, the initial onset of the pandemic brought on an exorbitant amount of stress and uncertainty for children and families [91]. As a result, it is possible that during Wave 1, the steep decline in mental health exacerbated existing conflicts among children and youth, or led to new challenges as they navigated changes in their typical routines and environments. Then, in subsequent waves, as they became more accustomed to the pandemic, rates of peer relationship difficulties began to decline. Alternatively, parents may have been so preoccupied with the vast adaptations required in their day-to-day lives, or they may not have been seeing these difficulties observationally, and thus issues surrounding peer relationships were not on their radar.

The frequency of peer relationship difficulties was significantly lower in Waves 2, 3, 4, and 5 in comparison to the pre-pandemic period. The rate of peer relationship difficulties for this sample declined during COVID-19. This is consistent with previous findings in which, compared to the pre-pandemic period, rates of aggressive behaviours, risk of harm to others, and interpersonal conflict decreased during the pandemic [86,92]. Without the social pressures of interacting with others at school or various afterschool programmes, some neurodiverse children and youth who typically experience more severe social difficulties may have been more relaxed while at home [93,94]. This sub-set of children and youth would have experienced a less negative impact than their same-age non-clinically referred counterparts.

### 4.2. COVID-19 and Peer Relationships Across Age Groups

Perhaps the most notable conclusion from this study was that across all three clinically referred age groups (4–7 years, 8–11 years, and 12–18 years), children and youth experienced fewer peer relationship difficulties during COVID-19, compared to the pre-pandemic period. Specifically, peer relationship difficulties reduced from 17.1% pre-pandemic to 8.4% during the pandemic for young children between the ages of 4–7 years. Similarly, children in middle childhood saw rates change from 23% pre-pandemic to 14.2% after the onset of the pandemic, and rates for adolescents changed from 19.2% pre-pandemic to 14.4% during. These findings are not surprising, aligning with the body of literature regarding the COVID-19 pandemic that highlights a significant decline in children’s social interactions during the height of the pandemic.

Additionally, it is important to note that this study utilized a clinical sample inclusive of children and youth seeking mental health treatment across Ontario. These individuals were experiencing mental health and behavioural challenges that likely impacted their peer relationships prior to the onset of the pandemic [95]. Other research has also found that for children and youth who had elevated internalizing or externalizing difficulties before the pandemic, the COVID-19 stay-at-home restrictions provided protective effects on their overall well-being (e.g., [96]).

Interestingly, despite the overall decline in peer relationship difficulties following the onset of COVID-19, children in middle childhood and those in adolescence were more likely to have conflict with peers compared to the youngest age group. These findings align with developmental literature, which suggests that adolescents are more likely to report higher rates of conflict, and the use of more cooperative resolution strategies compared to younger children, who utilize more aggressive tactics [97]. This trend could also be explained by the notion that during the lockdown period, the social relations of young children were supervised by others and were primarily restricted to close family members [24]. Thus, rates of peer conflict naturally declined due to the lack of opportunities to connect with peers at all.

### 4.3. Differences in Peer Relationships by Sex

Despite the inclusion of a larger female population across all timeframes in this study, males were more likely to have peer relationship difficulties during COVID-19. It is widely acknowledged in the literature that, whereas females typically report more relational issues with friends, and tend to use more conflict-mitigating strategies, peer conflict among males tends to be more overtly aggressive and hostile [97,98]. Furthermore, school-aged boys are more likely to be diagnosed with attention deficit hyperactivity disorder (ADHD) than school-aged girls [99]. The research suggests that these individuals lack appropriate social knowledge and socio-cognitive abilities, which contributes to greater peer difficulties [100] and peer dislike [101], and translates to fewer close friendships and greater peer rejection [102].

### 4.4. Patterns of Peer Relationship Difficulties Among Population Subgroups

Children and youth in the lower income quartiles experienced more peer relationship difficulties during the pandemic than those in the higher quartiles. After controlling for age, sex, and waves, individuals in Income Quartile 4 were less likely to have peer relationship difficulties, while there was no significant difference between those in Income Quartiles 1, 2 and 3. This aligns with previous research by Hjalmarsson [67], who found an association between relative household income and youth peer rejection. Other research has similarly found that children from less affluent households tend to have fewer friends and are more often isolated (e.g., [103]). Additionally, with school closures in effect, and thus limited access to school-based resources, those with lower socioeconomic backgrounds received less clinical services [104].

### 4.5. Other Factors Impacting Peer Relationship Difficulties

It is important to note that the model utilized in this study was only able to accurately predict 59.8% of children and youth who experienced peer relationship difficulties. Thus, it is crucial to consider other factors that may assist in identifying those who are struggling socially. For instance, considering whether a child has experienced bullying victimization could be impactful. Studies have found that victims of bullying are perceived by themselves, their peers, and teachers to have poorer social skills compared to non-victims [105], and both victims and bullies are less likely to adhere to social norms and expectations than those not involved in bullying [106]. Likewise, trauma could be another factor that would assist clinicians in identifying individuals with frequent social conflict. For example, adverse childhood experiences are negatively associated with peer relationship quantity and peer status [107]. Finally, it may be that the impact of parenting would explain the additional variance observed in this model. The parent–child relationship provides the foundation for children to develop many important social competencies, it allows them to safely examine features of the social world, and it provides a basis for solidifying expectations and assumptions about interactions with others [108]. Thus, differing parenting styles significantly impact peer relationship outcomes in middle childhood and adolescence.

### 4.6. Clinical Implications

Given the significance of healthy peer relationships, continuing to monitor these trends in the coming years will be of utmost importance, as the long-term effects of COVID-19 still remain largely uncertain [109]. Specifically, tackling this issue through a lens that incorporates equity, diversity, inclusion, and anti-racism will be necessary in order to reduce further inequities brought on by the pandemic.

Considering that children and youth in lower income households were most likely to experience peer relationship difficulties both pre- and during COVID-19, implementing programmes within the school setting would be strongly advised. Research suggests that school-based social-emotional learning intervention programmes are generally successful and produce positive effects for students overall [110]. Moreover, school-based intervention programmes have the capacity to reach all demographic groups and effectively ensure that all students can access available resources. This is especially significant given the increase in demand for mental health services, particularly in community health settings, as well as the shortage of qualified providers post-pandemic [111]. Providing intervention support in schools would thus reduce potential wait times and cost-related barriers for all.

Adjustments to policies and procedures should also be made in order to ensure that students of low-income families receive equitable distribution of devices and the internet to remain connected with their peers when school closures are in effect. Organizing socially distanced community events and activities in which students can interact face-to-face while still adhering to safety guidelines would also enhance social networks [112]. Schools could implement a Pen Pals programme (e.g., [113]), which would allow students to exchange letters with peers, thereby fostering peer connections while promoting literacy and communication skills. Offering comprehensive and effective social skills training programmes or workshops [114,115] for students who may be struggling with their peer interactions should be a priority. Should another pandemic arise, it will be crucial to prioritize equity and inclusivity by providing alternative means for social interaction to prevent further isolation and promote overall well-being for all children and youth [116].

### 4.7. Limitations

While this study highlights many important trends for children and adolescents post COVID-19, it is also not without its limitations. Data for this study were collected solely from mental health agencies across Ontario. Thus, the findings from this study may not be generalizable to individuals residing in other provinces or countries where the COVID-19 restrictions, school closures, and social and environmental factors likely differed. Similarly, this study only included children and youth who had been referred for mental health services and therefore our findings may not capture the social experience of other same-aged individuals who were not seeking mental health services. Also relevant, not only did referrals to agencies decline during COVID-19, but higher income families were more likely to be referred compared to lower income families during the pandemic, compared to the pre-pandemic period. Marginalized youth were also less likely to access services. Thus, the reduction in peer difficulties could be explained by the change in referral patterns, as lower income youth are more likely to exhibit behavioural problems [117]. Additionally, different types of children could have been referred prior to the pandemic; whereas pre-pandemic, schools would have referred children and youth with behaviour problems to agencies, during the pandemic, referrals were primarily from parents who were worried about their child’s mental health. Future studies should aim to explore this further.

Another limitation of this study is that we did not examine diverse sub-populations (single parenting, ethnicity, race, etc.). However, we did examine low income, which often is related to marginalized sub-populations as one of many indicators. Additionally, as data used in this study were only collected until March 2022, it is possible that trends in peer relationships have continued to change over time and investigation into more recent patterns is warranted. Moreover, given the cross-sectional nature of this study, no causal claims can be made. Since individuals were not studied longitudinally, our understanding of an individual’s complete social experience is still limited. Finally, this study utilized archival data, which did not allow us to investigate other factors beyond the individual both pre-pandemic and during COVID-19, such as varied cultural experiences, specific periods of school closures, individual school and school board rules and regulations, and peer group dynamics.

### 4.8. Future Directions

Given that our study only captures the experience of children and youth residing in Ontario, future research should be conducted with clinically referred children worldwide to improve the generalizability of the study findings. Longitudinal investigations are also important to understand potential long-term impacts of the pandemic. For example, studying “sleeper” effects related to the lack of exposure to peer relationships in early childhood could be conducted. Similarly, examining how the period of social isolation may have impacted future social competence because fundamental skills (i.e., turn-taking, sharing, etc.) were not practised during the pandemic.

## 5. Conclusions

The present study builds upon the available literature by examining differences in social relationships pre- versus during COVID-19 for children and youth across various age groups. Our findings revealed that overall peer relationship difficulties declined over the course of the pandemic, beginning in Wave 2 and remaining low through Wave 5 for clinically referred children and youth. Specifically, older children and adolescents, males, and those in the lower income quartiles were more likely to experience peer relationship difficulties than others. Investigating unique peer relationship patterns is also needed to understand the impact of the pandemic on certain subgroups who may be in most need for social and behavioural interventions.

## Figures and Tables

**Table 1:** Descriptive Statistics for Demographic Characteristics.

Variable	Pre-COVID	Wave 1	Wave 2	Wave 3	Wave 4	Wave 5
	*N* (%)	*N* (%)	*N* (%)	*N* (%)	*N* (%)	*N* (%)
Age						
4–7 years	674 (12%)	161 (15.1%)	198 (13.2%)	145 (11.9%)	125 (11.2%)	93 (12%)
8–11 years	1485 (26.5%)	265 (24.9%)	370 (24.8%)	310 (25.4%)	259 (23.2%)	229 (29.4%)
12–18 years	3449 (61.5%)	638 (60%)	927 (62%)	764 (62.7%)	733 (65.6%)	456 (58.6%)
Sex						
Male	2903 (51.8%)	493 (46.3%)	645 (43.1%)	498 (40.9%)	465 (41.6%)	357 (45.9%)
Female	2705 (48.2%)	571 (53.7%)	850 (56.9%)	721 (59.1%)	652 (58.4%)	421 (54.1%)
Area median household income quartile after tax						
<$57,367	1564 (27.9%)	238 (22.4%)	333 (22.3%)	267 (21.9%)	234 (21%)	194 (24.9%)
$57,367 to $70,334	1133 (20.2%)	191 (18%)	281 (18.8%)	210 (17.2%)	208 (18.6%)	155 (19.9%)
$70,335 to $84,750	1497 (26.7%)	318 (29.9%)	464 (31%)	402 (33%)	391 (35%)	243 (31.2%)
> $84,750	1414 (25.2%)	317 (29.7%)	417 (27.9%)	340 (27.9%)	284 (25.4%)	186 (23.9%)

**Table 2:** Pearson Chi-Square Analyses Pre-COVID.

Variable	No Peer Relationship Difficulties *N* (%)	Peer Relationship Difficulties *N* (%)	χ^2^	*p*	Cramer’s *V*
Age			13.008	0.0015	0.048
4–7 years	559 (82.9%)	115 (17.1%)			
8–11 years	1144 (77%)	341 (23%)			
12–18 years	2785 (80.8%)	664 (19.2%)			

**Table 3:** Pearson Chi-Square Analyses During COVID.

Variable	No Peer Relationship Difficulties N (%)	Peer Relationship Difficulties N (%)	χ^2^	*p*	Cramer’s *V*
Age			18.442	<0.0001	0.057
4–7 years	661 (91.6%)	61 (8.4%)			
8–11 years	1230 (85.8%)	203 (14.2%)			
12–18 years	3013 (85.6%)	505 (14.4%)			

**Table 4:** Model Fit Information.

Model	Likelihood Ratio	*p*-Value	*C* Statistic	*R*-Square
Model 1	101.900	<0.0001	0.567	0.009
Model 2	127.560	<0.0001	0.578	0.011
Model 3	160.222	<0.0001	0.589	0.014
Model 4	190.486	<0.0001	0.598	0.017

**Table 5:** Hierarchical regression analysis: peer relationship difficulties as a function of wave, age, sex, and household income.

Model	Predictor	*B*	Wald Chi-Square	Odds Ratio Exp(B)	99% C.I.	*p*-Value
Model 1	Wave					
	Wave 1 ^a^	−0.199	4.980	0.821	0.615, 1.098	0.026
	Wave 2 ^a^	−0.407	25.110	0.666	0.510, 0.870	<0.0001
	Wave 3 ^a^	−0.654	46.575	0.520	0.379, 0.713	<0.0001
	Wave 4 ^a^	−0.523	30.040	0.593	0.433, 0.812	<0.0001
	Wave 5 ^a^	−0.633	29.586	0.531	0.362, 0.779	<0.0001
Model 2	Wave					
	Wave 1 ^a^	−0.186	4.421	0.831	0.621, 1.111	0.036
	Wave 2 ^a^	−0.402	24.436	0.669	0.512, 0.874	<0.0001
	Wave 3 ^a^	−0.654	46.541	0.520	0.379, 0.713	<0.0001
	Wave 4 ^a^	−0.522	29.894	0.593	0.434, 0.812	<0.0001
	Wave 5 ^a^	−0.634	30.024	0.528	0.360, 0.775	<0.0001
	Age					
	Ages 8–11 ^b^	0.464	24.326	1.590	1.167, 2.166	<0.0001
	Ages 12–18 ^b^	0.340	15.212	1.405	1.055, 1.871	<0.0001
Model 3	Wave					
	Wave 1 ^a^	−0.168	3.606	0.845	0.632, 1.131	0.058
	Wave 2 ^a^	−0.377	21.439	0.686	0.524, 0.897	<0.0001
	Wave 3 ^a^	−0.623	41.961	0.536	0.391, 0.736	<0.0001
	Wave 4 ^a^	−0.495	26.789	0.610	0.445, 0.835	<0.0001
	Wave 5 ^a^	−0.621	28.277	0.538	0.366, 0.789	<0.0001
	Age					
	Ages 8–11 ^b^	0.480	25.975	1.616	1.185, 2.202	<0.0001
	Ages 12–18 ^b^	0.419	22.543	1.521	1.137, 2.034	<0.0001
	Sex ^c^	−0.299	32.573	0.742	0.624, 0.881	<0.0001
Model 4	Wave					
	Wave 1 ^a^	−0.147	2.742	0.863	0.645, 1.156	0.098
	Wave 2 ^a^	−0.361	19.553	0.697	0.533, 0.912	<0.0001
	Wave 3 ^a^	−0.604	39.300	0.547	0.398, 0.750	<0.0001
	Wave 4 ^a^	−0.481	25.155	0.618	0.451, 0.848	<0.0001
	Wave 5 ^a^	−0.620	28.095	0.538	0.366, 0.791	<0.0001
	Age					
	Ages 8–11 ^b^	0.498	27.886	1.646	1.207, 2.245	<0.0001
	Ages 12–18 ^b^	0.435	24.112	1.545	1.154, 2.068	<0.0001
	Sex ^c^	−0.299	32.494	0.742	0.624, 0.881	<0.0001
	Household Income					
	2nd quartile ^d^	−0.008	0.012	1.008	0.791, 1.284	0.914
	3rd quartile ^d^	−0.178	6.828	0.837	0.668, 1.047	0.009
	4th quartile ^d^	−0.339	22.252	0.712	0.562, 0.903	<0.0001

Note: C.I.—confidence interval. ^a^ In reference to the pre-pandemic period. ^b^ In reference to age 4 to 7-year-olds. ^c^ In reference to the male sex. ^d^ In reference to the 1st income quartile.

## Data Availability

Due to the sensitive nature of this study, participants were assured their data would remain confidential and would not be shared.
